# Injuries in alpine summer sports - types, frequency and prevention: a systematic review

**DOI:** 10.1186/s13102-022-00468-4

**Published:** 2022-05-01

**Authors:** Sebastian Frederick Bigdon, Verena Hecht, Paul Gilbert Fairhurst, Moritz C. Deml, Aristomenis K. Exadaktylos, Christoph E. Albers

**Affiliations:** 1 Department of Orthopaedic Surgery and Traumatology, Inselspital, University Hospital, University of Bern, Freiburgstrasse, 3010 Bern, Switzerland; 2 Department of Emergency Medicine, Inselspital, University Hospital, University of Bern, Freiburgstrasse 16C, 3010 Bern, Switzerland

**Keywords:** Alpine sport, Airborne sport, Mountain bike, Trauma, Alpine injuries, Summer sports

## Abstract

**Introduction:**

Summer alpine sports, including mountain biking, hiking and airborne pursuits, have experienced a recent surge in popularity. Accordingly, trauma associated with these activities has increased. There is a scarcity of literature exploring clinical aspects surrounding injuries. Specifically, no single article provides a general overview, as individual studies tend to focus on one particular sport. In the present study, we performed a systematic literature review to summarize existing knowledge and explore the potential for prevention and clinical decision making in this group.

**Method:**

Literature searches were performed using the PubMed and Scopus database for the most commonly ventured sports associated with injury: mountain biking, climbing, airborne sports, paragliding, and base jumping. From this search, studies were identified for qualitative and quantitative analyses. These searches were done according to PRISMA guidelines for systematic reviews. Studies were then analyzed regarding epidemiology of injuries, relevant anatomical considerations and prevention strategies were discussed.

**Results:**

A broad spectrum of injury sites and mechanisms are seen in mountain biking, climbing or airborne sports. Mountain biking related injuries commonly involve the upper extremity, with fractures of the clavicle being the most common injury, followed by fractures of the hand and wrist. Scaphoid fractures remain of paramount importance in a differential diagnosis, given their often subtle clinical and radiological appearance. Paragliding, skydiving, and base jumping particularly affect transition areas of the spine, such as the thoracolumbar and the spinopelvic regions. Lower limb injuries were seen in equal frequency to spinal injuries. Regarding relative risk, mountain biking has the lowest risk for injuries, followed by climbing and airborne sports. Male alpinists are reported to be more susceptible to injuries than female alpinists. Generally, the literature surrounding hiking and water-related mountain sports is insufficient, and further work is required to elucidate injury mechanisms and effective preventative measures. A helmet seems to decrease the likelihood of face and head injuries in mountain sports and be a meaningful preventive measurement.

## Introduction

With the increase in popularity for a wide variety of sport and leisure activities in the Alps during the summer months, such as Mountain Biking, Hiking and airborne, there has been an increase in trauma incidence associated with these activities [1]. Detailed knowledge of injury patterns, frequency, mechanisms, and risk factors is therefore of great importance to prevent injuries and act correctly in clinical management. However, no single review has encompassed such injury patterns in the literature. We therefore performed a literature review to summarize existing knowledge and explore the potential for improved prevention and clinical decision making in this context.

In an analysis of alpine accidents and injuries in 2020, the Swiss Alpine Club (SAC) was involved in rescuing 3471 people [1]. The year before, only 2909 people had accidents. That is almost 20% more than the previous year [2].

Around one-third of the injuries described occurred during hiking, followed by mountain biking, paragliding and rock climbing. Most of these cases resulted in hospitalization and required medical or surgical intervention [1].

Looking at mountain biking in more detail, more than 30 million people practise this sport worldwide [3]. The “Union Cyclist International” the defined most common types are cross country, downhill and marathon [4]. Popularity is increasing every year, so much so that in the United States alone, the number of mountain bikers has risen from 6.75 million in 2006 to 8.6 million in 2017 [5, 6].

Taking a closer look at climbing, the most common types are lead climbing, speed climbing and bouldering [7]. In the last decade, indoor climbing has become an increasingly popular sport worldwide. This trend will most likely continue as it has recently been included in the Olympic program (Tokyo 2020) [8].

Regarding airborne sport, there are three major subtypes, which are paragliding, base jumping and skydiving on the one hand [9, 10]. Since 1905, the increasing number of airborne sports has been accompanied by an increasing number of people engaging in these sports, and the number of related injuries has also gradually increased [11].

This review is intended to provide an overview of alpine sports activities in summer and the injuries associated with them. This should help guide the treating physician with valuable information for subsequent clinical management and possible preventive factors.

## Methods

Relevant studies were reviewed based on systematic review (PRISMA) guidelines. [12]

### Eligibility criteria

Observational, cohort, epidemiological studies assessing the incidence and prevalence of trauma, visceral or orthopaedic injuries during mountain biking, climbing, paragliding, base-jumping and skydiving were included in the study. Prospective and retrospective studies with professionals and amateurs over 18 years were eligible and included. No date limitation was applied to the studies. Articles written in English, German or professionally translated into English were included.

### Outcome measures

The primary outcome measures were the incidence or prevalence of injuries in alpine summer sports (mountain biking, climbing, paragliding, base jumping, skydiving). Secondary outcome measures included the severity and the location of the reported injuries and evaluation of preventive measurements.

### Data sources and search strategy

**Fig. 1:**
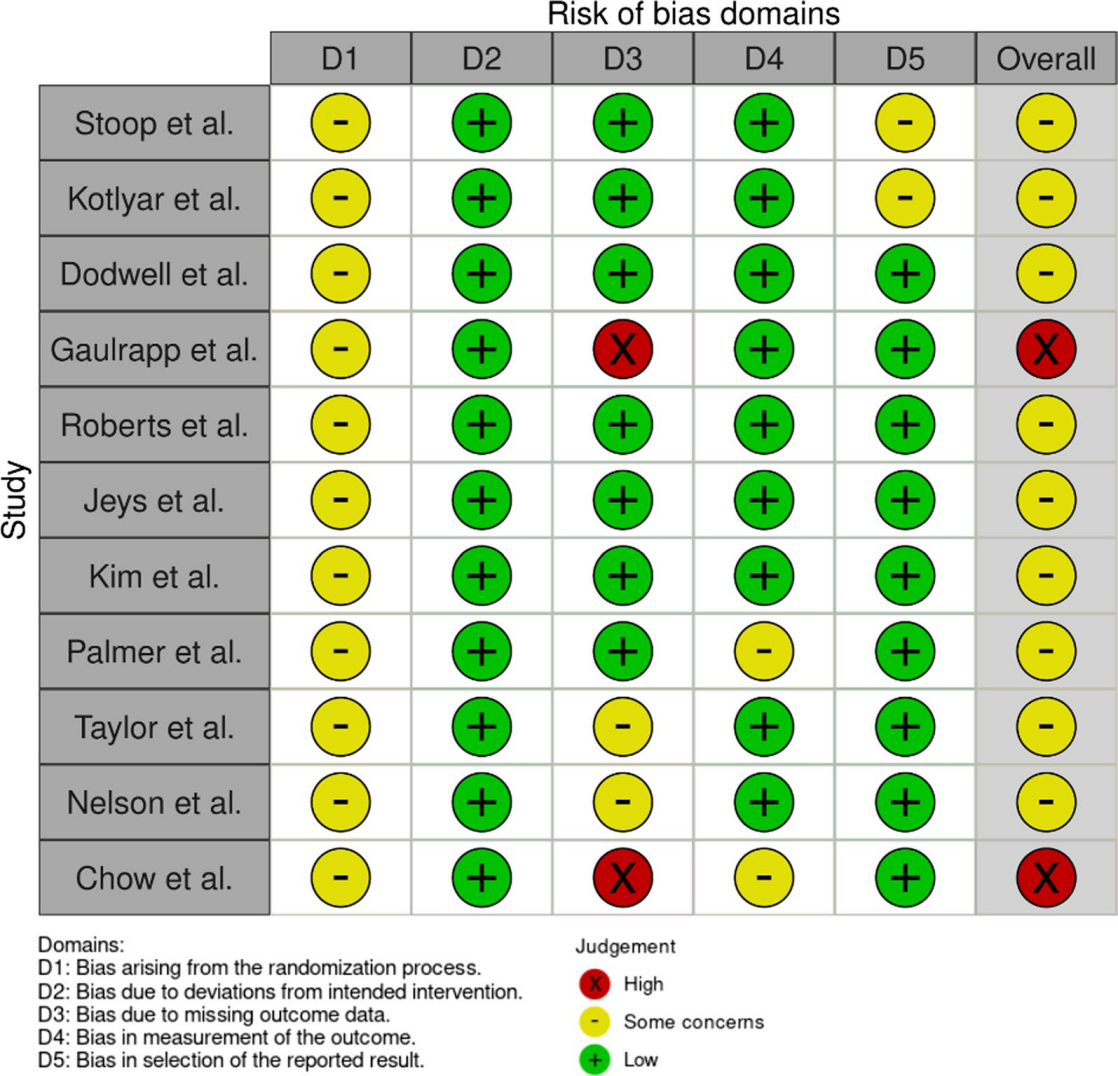
Risk-of-bias assessment for the studies concerning mountain biking using the RoB 2 tool, visualized with the use of robvis. All of the studies showed at least some concern for bias

**Fig. 2:**
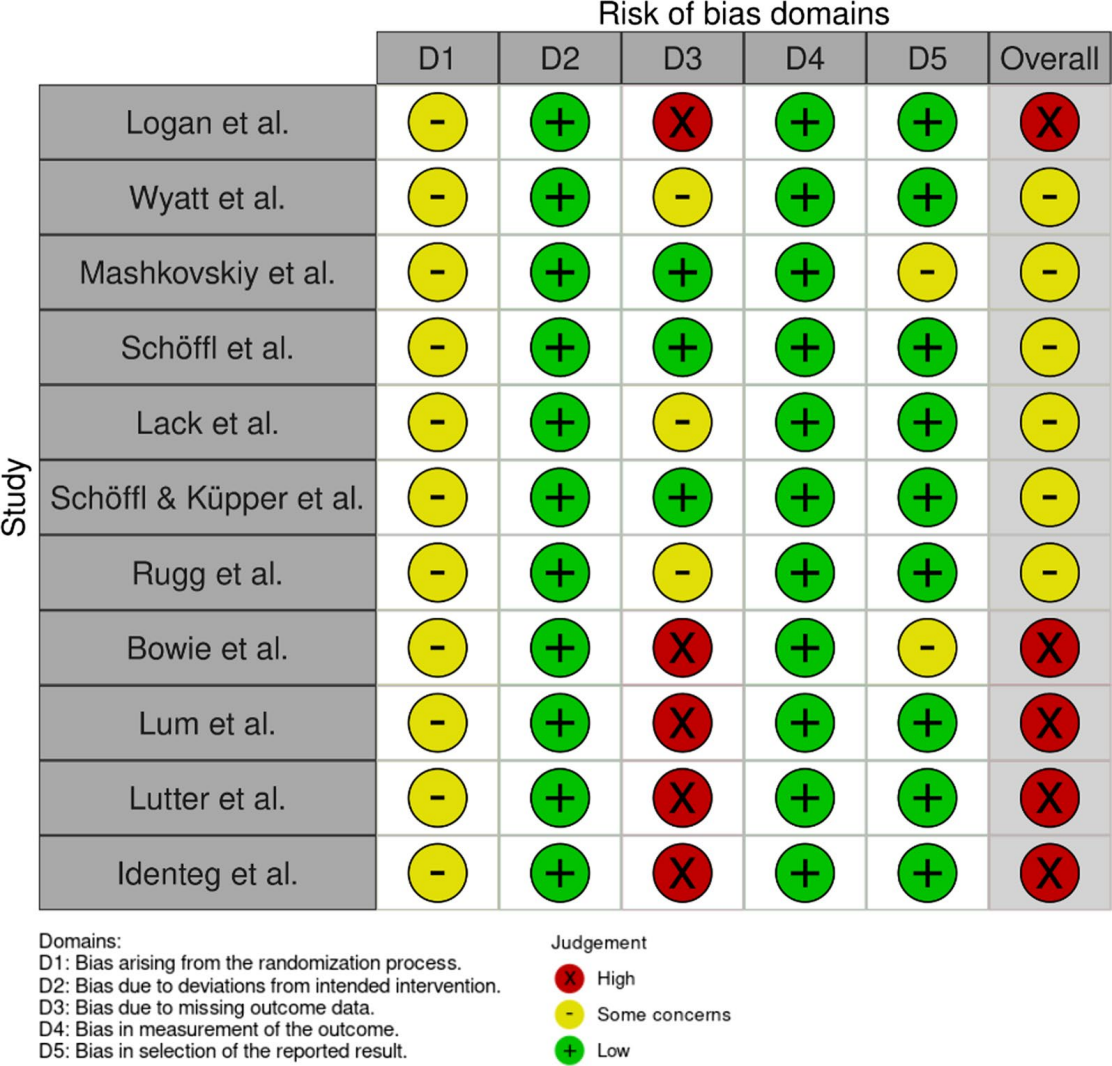
Risk-of-bias assessment for the studies concerning climbing using the RoB 2 tool, visualized with the use of robvis. All of the studies showed at least some concern for bias

**Fig. 3:**
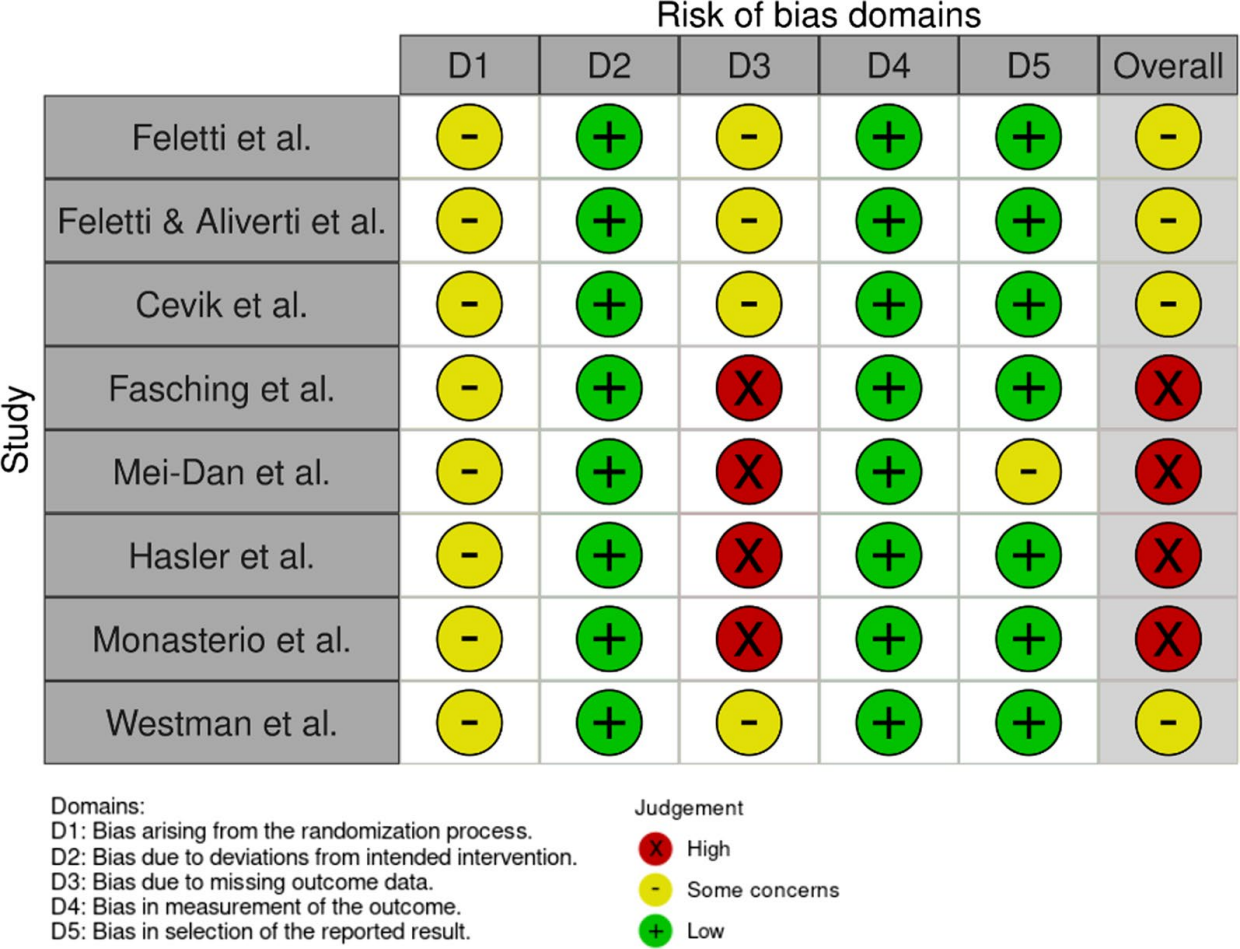
Risk-of-bias assessment for the studies concerning airborne sports using the RoB 2 tool, visualized with the use of robvis. All of the studies showed at least some concern for bias

PubMed and Scopus Databases were searched by VH and SFB. The reference lists of eligible articles, identified during the search, were manually searched. Databases were searched using the following keywords. For mountain biking “Mountain biking, injuries [MAJR]” was used, for climbing “Climbing, injuries [MAJR]”, for paragliding “paragliding, injuries [MAJR]”, for base jumping “base jumping, injuries [MAJR]” and for skydiving “skydiving, injuries [MAJR]”. The database searches took place on 18 November 2021 and included all relevant publications up to this date (Figs. 1, 2, 3).

**Fig. 4:**
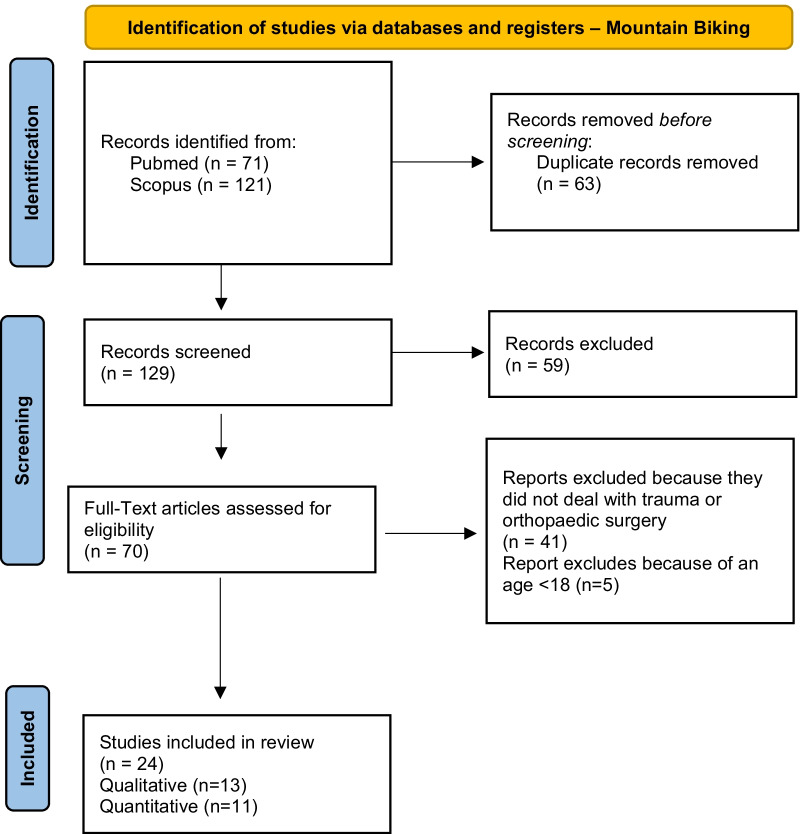
Flow diagram for study inclusion and exclusion in Mountain biking

**Fig. 5:**
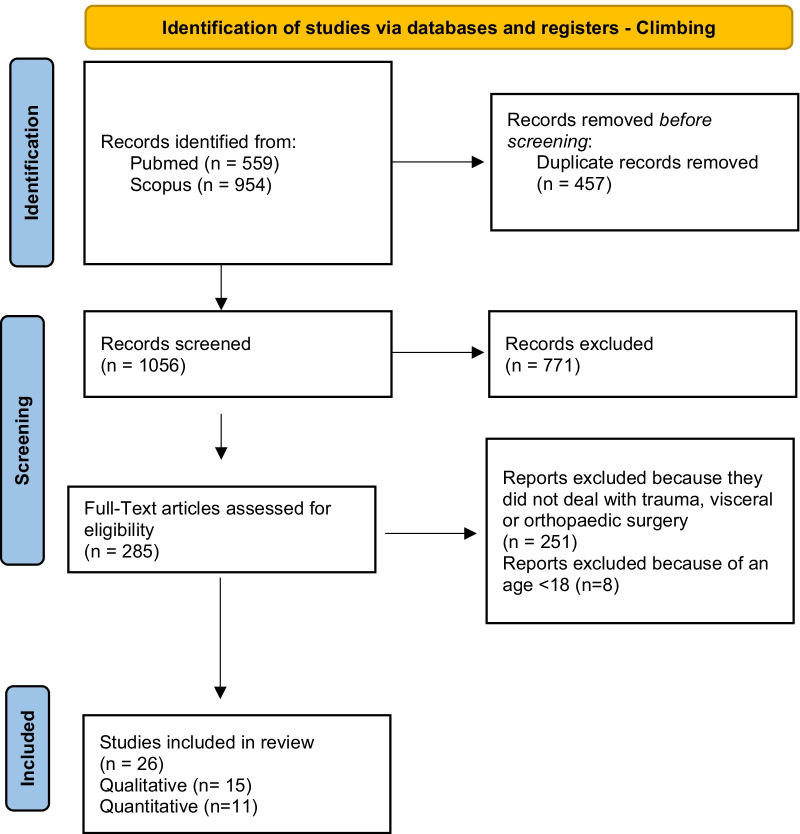
Flow diagram for study inclusion and exclusion in climbing

**Fig. 6:**
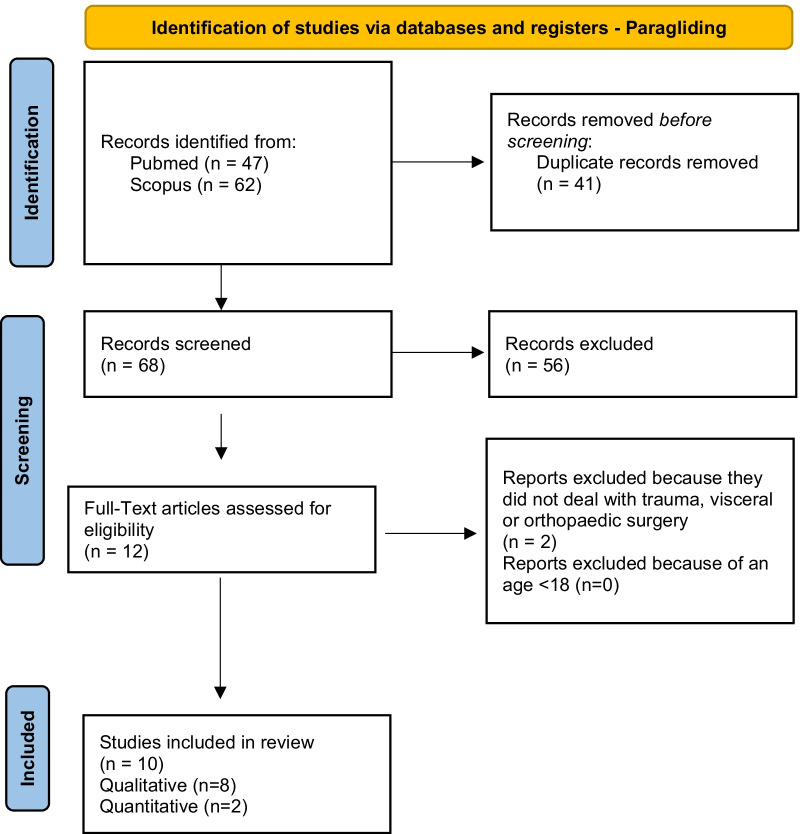
Flow diagram for study inclusion and exclusion in paragliding

**Fig. 7:**
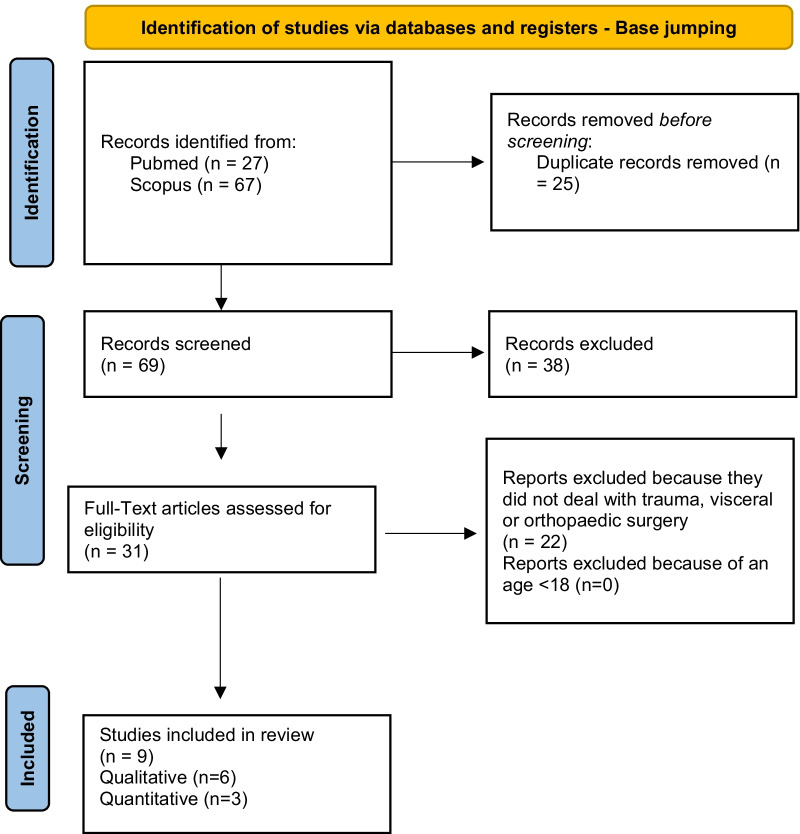
Flow diagram for study inclusion and exclusion in Base jumping

**Fig. 8:**
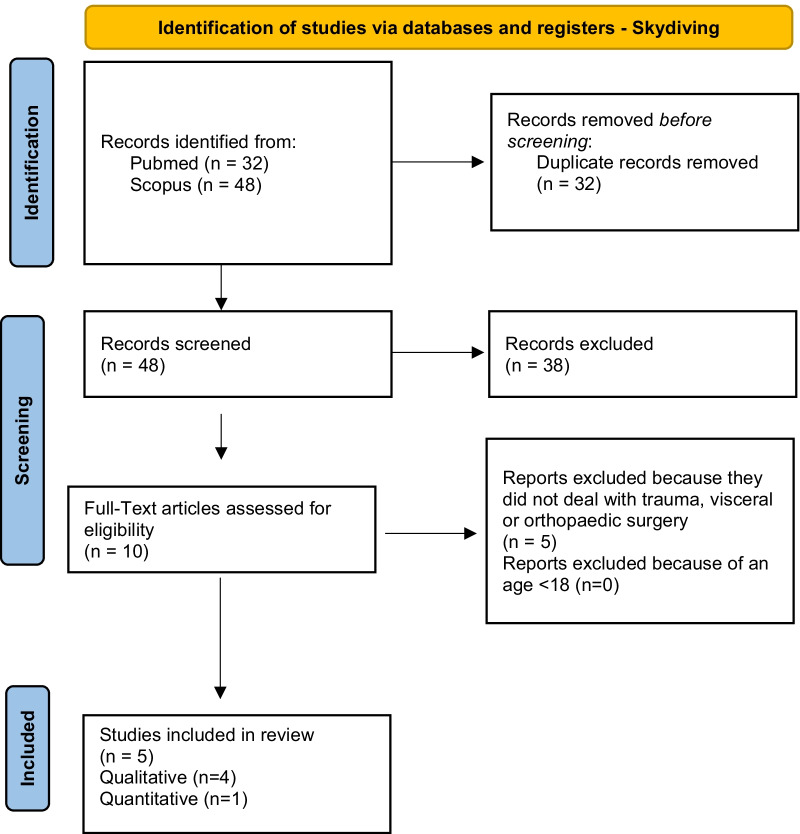
Flow diagram for study inclusion and exclusion in Skydiving

According to PRISMA guidelines, the search strategy was recorded in Figs. 4, 5, 6, 7 and 8 [12].

### Data screening and extraction

We (VH and SFB) screened the title and abstracts for eligibility based on the criteria as mentioned earlier independently. Once the articles were included, we extracted the appropriate data from the text. Data were extracted by the reviewers independently, on participants (age and sex), number of participants and injuries, injury location and percentage. A qualitative evaluation has been performed.

### Risk of Bias assessment

The revised Cochrane risk-of-bias tool (RoB 2) for randomized trials was used to assess the Risk of Bias [13]. Two authors (VH and SFB) independently conducted the Risk of Bias assessment. Discrepancies were resolved by consensus or by third-party arbitration (CA). The Risk of Bias assessment strictly followed the recommendations provided by the RoB 2 tool and included five key domains: (D1) bias arising due to the randomization process, (D2) bias due to deviation from the intended intervention, (D3) bias due to missing outcome data, (D4) bias in the measurement of the outcome, and (D5) bias in the selection of the reported result. These results were visualized with the robvis visualization tool [14].

### Quality of reporting

Reporting quality assessment included questions on whether the aims, population and methods were clearly reported in the article. Results and limitations need to be adequately described and discussed.

### Quality of design

The quality of design was evaluated on its appropriateness for the study's aims and the justification of the sample size and frame. Conflicts of interest and ethical approval were assessed in this section.

### Data synthesis

A quantitative representation using descriptive tables, without doing further statistics, would have been performed if studies had reported sufficient data in the same format or with the same definitions of injuries and percentages of injury locations. Descriptive summary tables were populated with information from each study, including investigation period, participants (sex, mean age, number of participants) and outcomes (injury location). All injury data were reported as an incidence (per time period) or prevalence (percentage). If the study may not have qualified for a quantitative representation, it was used qualitative.

## Results—mountain biking

### Epidemiology

The incidence of injuries in mountain biking has an overall injury risk rate of 0.6% per year and between 0.52 up to 16.8 injuries per 1000 h of mountain biking [15–17]. Stoop et al. and Gaulrapp et al. also showed that the prevalence of injuries does not differ between amateurs and elite athletes [17, 18]. In case of races injury rates are higher with about 43.4 injuries per 1000 h downhill racing. [19] Serious injuries, defined as limb or life-threatening, occur with 5 per 1000 h of downhill riding [6]. A retrospective study of cross country riders found that 90% of riders reported at least one injury during the season, with 10% being “serious” [18]. The mortality rate is about 0.0035 per 1000 persons at risk [20].

Age at injury ranged from 25 to 40.7 years [17, 18, 21]. The male to female ratio ranged from 2:1 to 49:1 [18, 21–23]. The severity of the injury was significantly lower for female versus male riders. [15]

Injuries occurred in the mountain bike park 73% of the time, followed by trails (18%) and hills (5%). At the time of the accident, most riders were riding downhill (60%) on steep and rocky stages [5, 15, 24].

65% of the injuries happened between 12 and 6 pm [21]. 44% mentioned slippery terrain as an accident cause, 34% reported situational judgement errors and 33% excessive speed [18].

### Injury type and severity

The most common injuries were orthopaedic injuries (46.5%), and 68–88.5% were fractures, followed by ligamentous injuries and instabilities [25, 26].

The majority of injuries were not severe or life-threatening. Only 10% of injuries required cessation of activity for more than three weeks or an immediate [18]. Falls over the handlebars are the most common injury mechanism. Consequently, the head, torso, and upper extremities are more commonly injured, especially in amateur riders, as seen in Table 1 [3, 17, 18, 21, 22, 27–29]. Overall, the hand and shoulder are most susceptible to injury. Clavicle fractures occur with the highest frequency (17.5%-62.5%) [26, 27, 30, 31]. Concerning hand injuries, fractures of the fingers are also prevalent (22.9%) [18], as well as fractures of the metacarpale (26%) [24]. Regarding overuse injuries of the upper limb, wrist neuropathy is common due to the constant vibration of the handlebars [19].

**Table 1:** Injury region of injuries in mountain biking, whereas “n.r.” means not reported, “m:f” means male:female

Author	Investigation period	m:f (%) mean age	Number of injuries	Number of injured riders	Amateurs/Elites	Type of injury	Injury location							
							Upper extremity			Lower extremity			Trunk and spine	Head and neck
							Shoulder	Hand/finger	Upper/lower arm	Hip/thigh	Knee/calf	Foot/ankle		
Stoop et al. [17]	2017	n.r. 32.5	n.r	15	Amateurs and elites	Fractures and joint injuries	13.4%	15.7%	6.7%	19.3%	34.6%	3.1%	5%	2.2%
Kotlyar et al. [22]	2012—2015	70:30 n.r	n.r	304	Amateurs	all	76.2%	4.8%	8.3%	10.7%				
Dodwell et al. [21]	1995—2007	95:5 32.7	107	n.r	Amateurs except 2 elites	Spinal injuries	n.r	n.r	n.r	n.r	n.r	n.r	Cervical: 73.8% Thoracic/lumbar: 26.2%	n.r
Gaulrapp et al. [18]	n.r	98:02 25	3873	n.r	Amateurs and elites	All	8.6%	15.2%	22.0%	12.8%	23.0%	3.0%	6.3%	9.1%
Roberts et al. [32]	1995–2009	87:13 39	99	49	Amateurs	All		6.7%			6.6%		51%	35.7%
Jeys et al. [30]	n.r	71:29 22.5	133	84	n.r	All	75.7% (clavicle: 17.5%)	5.8%	3.9%	14.6%				
Kim et al. [25]	1992–2000	n.r. n.r	399	1092	n.r	All	25%	29%	27.7%	12.2%				
Palmer et al. [15]	2017–2018	90:10 n.r	188	179	Elites	All	37.2%	26.1%	6.4%	12.2%				
Taylor et al. [23]	2000–2007	88:12 n.r	595	596	Elites	All	59.2%	3.2%	10.1%					
Nelson et al. [27]	1994–2007	81:19 29.8	-	4624	Amateurs	All	26.9%	n.r	n.r	19.6%	n.r	n.r		
Chow et al. [28]	–	83:17 36.2	437	225	Amateurs	All	34.3%	36.4	8.9%	20.4%				

Spinal injuries do appear scarcely. Dodwell et al. showed a spinal injury rate of 0.20/100 000 participants. The cervical spine was most commonly injured (78.3% of spinal injuries), predominantly the middle and lower cervical spine (C3-7 at 82.3%). 40% of patients with spinal injuries sustained a spinal cord injury with neurologic disability [21].

Head injury frequency ranged from 2.2 to 35.7% in various studies [17, 18, 22, 32].

Another injury mechanism is falling against the handlebars. This causes especially intraabdominal traumata with liver hematomas [33]. Kotlyar et al. described abdominal injuries in only 1% of all injuries on racing trails [22].

Lower extremity injuries, which occur less frequently, are often attributed to injuries related to bicycle contact, especially with bicycle pedals or a lateral fall. [17, 18, 27].

### Prevention

Helmets are the most frequently reported preventive measure for mountain biking. The reported helmet use varies widely in the literature. Chow et al. found that 88% of mountain bikers in the United States wore a helmet at the time of the accident, whereas Kotlyar et al. showed that less than 50% routinely wear helmets in mountain biking in the United States [22, 34]. Dodwell et al. and Gaulrapp et al., reported much higher rates of helmet use (83–85.6%) [18, 21]. However, Stoop et al. found no correlation between protective gear use and overall injury frequency (r = − 0.180, *p* = 0.521) [17]. Although Dodwell et al. showed that the mean Injury Severity Score (ISS) for helmet compliant and non-compliant groups is not different (16.4:16.3) [21]. In contrast to that, helmets worn by mountain bikers have been shown to be effective in reducing facial and head injuries [19, 34].

Kotlyar et al. showed that patients who did not wear helmets were more likely to require transfer to a neurosurgical unit (38% vs 17%; *p* = 0.296) [22].

The most frequently worn protection equipment after a helmet are gloves (87–90%), knee protection (76–96%) and back protection (54–82%) [35].

## Results—climbing

### Epidemiology

In climbing, there are two common injury types: overuse syndromes caused by repetitive hand and finger stress, and acute injuries caused by falls or falling rocks [36, 37]. Overuse injuries occur more often than injuries caused by falls [38].

About 75% of climbers suffer an upper extremity overuse syndrome once in their career [36]. To understand the common overuse injuries, it is crucial to understand the basic grip positions. The pocket grip involves inserting one or two fingertips into a small pocket in the rock, with considerable stress on the flexor tendons [39]. The crimp grip involves four fingers in a handhold, with proximal interphalangeal joints (PIP) flexed at 90 degrees and distal interphalangeal joints (DIP) in full extension. The joint position is the most crucial factor determining injury patterns [36, 39]. A pinch grip is used for gripping small outcrops. This grip is associated with pain in the metacarpal-phalangeal joint (MCP). Crack climbing involves supporting one’s body weight with one finger. This is associated with fingertip injuries and amputations [39].

Ice climbing is a unique form of climbing and has one of the highest injury rates in climbing, with 9.8 injuries per 1000 h of exposure [40]. Indoor climbing by stark contrast was shown to lead to 3.1 injuries per 1000 h of exposure [41]. Fatal injuries occurred in 4.7% of all cases and they mainly occurred after a fall with ground impact [37].

The mean age at injury of climbers was 29–50 years [42–44]. The male to female ratio ranged from 4:1 to 10:1 [42, 44, 45]. One study investigated injuries at rock-climbing world championships, which showed a male to female ratio of 0:100, with only four injuries reported [41].

A study by Lack et al. showed that the highest number of victims occurred during unroped climbing [45].

The injury rate per 1000 climbing hours was 0.19 for men and 0.23 for women [46]. Men had higher proportions of ligament injuries of the fingers, whereas women had higher proportions of ligament injuries of the feet [46].

### Sites and types of injury

**Fig. 9:**
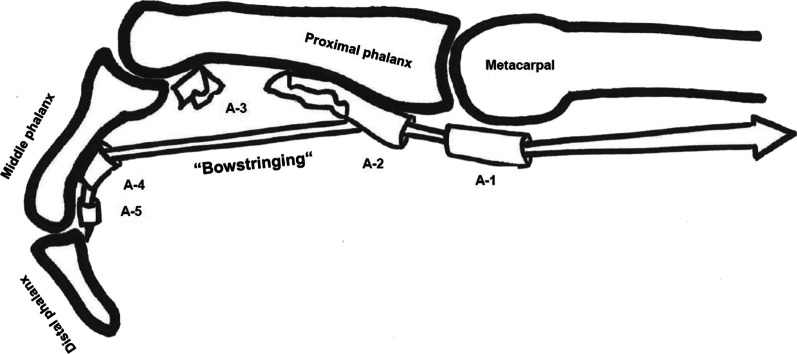
Mechanism of Bowstringing injury (own illustration) modified according to Neumann, Kinesiology of the Musculoskeletal System: Foundations for Rehabilitation [25]

As in mountain biking, most injuries involved the upper extremities, as seen in Table 2 [43, 47]. However, fractures were more unlikely and the most common site for injuries were the fingers (48.7%-65.1) [8, 43, 48], especially because of overuse [36, 42, 43] or falling while holding onto small holds [49]. Of these, the most common were tendon injuries [42], especially the flexor tendons or the annular ligaments [8, 43, 47], often causing so-called pulley injuries (for illustration see Fig. 9) [39, 47]. These pulley injuries can occur chronically or acutely [50]. The pulley system in the fingers is crucial in maintaining contact of digital flexor tendons with the underlying bone. Such injuries involve the annular and cruciate ligaments of the fingers. The most significant structures to prevent bowstringing biomechanically speaking are the A2 and A4 annular ligaments (Fig. 9) [36, 39, 51]. Many studies show that these are the pulleys, which first rupture [36, 39]. Of all finger injuries, 30% were pulley injuries [8, 43]. The most affected finger is the ring finger [52] or the middle finger [49]. The average probability of sustaining at least one reinjury due to climbing is 35.6%, especially in fingers [53].

**Table 2:** Injury region of injuries in climbing, whereas “n.r.” means not reported, “m:f” means male:female

Author	Investi-gation period	m:f (%) mean age	Number of injuries	Number of injured riders	Amateurs/elites	Type of injury	Injury location							
							Upper extremity			Lower extremity			Trunk & Spine	Head & Neck
							Shoulder	Hand/finger	Upper/lower arm	Hip/thigh	Knee/calf	Foot/ankle		
Logan et al. [42]	n.r	91:09 50	n.r	545	Amateurs and elites	Hand and finger injuries	n.r	28%	n.r	n.r	n.r	n.r	n.r	n.r
Wyatt et al. [44]	1992–1993	79:21 29	19	n.r	n.r	all	n.r	21%	n.r	0%	26%	37%	16%	n.r
Mashkovskiy et al. [56]	2014	n.r. n.r	21	n.r	Amateurs	All	n.r	n.r	5%	33%	14%	n.r	5%	43%
Schöffl et al. [8]	2009—2012	n.r. 34.1	911	n.r	Amateurs and elites	All	17.2%	65.1%	9.1%	0.4%	2.1%	3.8%	1.9%	n.r
Lack et al. [45]	1998 -2011	78:22 n.r	n.r	242	Amateurs and elites	All	2%	2.5%	29.5%	16.5%	17%			
Schöffl et al. [8]	2005	90:10 n.r	4	n.r	Elites	All	n.r	n.r	n.r	n.r	50%	25%	25%	n.r
Rugg et al. [37]	2005–2018	79:21 n.r	1503	1450	Amateurs	All	8.7%	9.4%	2.2%	4.5%	10.9%	27.1%	0.4%	21.2%
Bowie et al. [57]	1984–1987	88:12 n.r	451	220	Amateurs	All	2%	12%	2%	10%	12%	54%	4%	4%
Lum et al. [55]	2017–2018	83:17 27.4	432	237	Amateurs	All	17.3%	41.9%	2.8%	7.4%	19.9%	3.7%	n.r	
Lutter et al. [48]	2017–2018	69:31 30.8	633	436	Amateurs	All	18.2%	48.7%	10%	1.7%	8.2%	8.6%	2.8%	0.7%
Identeg et al. [54]	2008–2019	79:21 n.r	38	38	Amateurs	All	5%	5%	3%	66%	13%	3%

The study of Nelson et al. showed that as the level of experience increases, the odds of finger injury also increase (beginners 0.19 (95% CI, 0.01–4.64), leisure climbers 0.18 (95% CI, 0.04–0.78), amateur climbers 0.26 (95% CI, 0.06–1.20)) [47].

Injuries to the spine occurred in 1.9 to 16.5% of all injuries [43–45, 54, 55]. However, lower extremity injuries were also commonly reported at 6 to 74% of all injuries [43, 44, 54, 56, 57]. Injuries caused by falling mostly involve the ankle or the foot (27.1–66%), whereas injuries caused by falling rocks mainly involve the head (3–21.2%) [37, 54]. Ankle injuries were also frequently reported (4 to 66% of all injuries) [43, 44, 54], primarily because of falling from a high height in forced supination, because of the small climbing shoes [36, 41, 43, 44, 55]. Ankle injuries were more reported in females than in males [58].

Mashkovskiy et al. showed that amateurs mostly sustained cranial injuries, while thigh injuries were most common in elite climbers [56].

Most accidents happened at the weekend and the median incident time was 3:30 [45].

A swiss study of Hasler et al. showed that a risk factor for injuries is climbing experience > 10 years and no previous experiences of the climbing route [59].

### Prevention

A suggested option for preventing overuse injuries in fingers is prophylactically taping the fingers around the proximal and middle phalanx base. In an experimental setup, it was shown that the tape (zinc oxide tape), when it has 2.5 turns and is 1.5 cm wide, can absorb forces of 500 N, and thus absorb large forces to which the tendons and pulleys are subjected during crimp grip [36].

Taping and weight training reduces injury rates in sport climbing and bouldering [60].

Ankle sprains can be reduced in covering the entire potential landing area with energy-absorbing materials [36].

Severe or fatal injuries were more probable when not wearing a helmet (OR 1.81; CI 1.35–2.43), with the main injury localization being the head [37].

There are no associations between climbing related chronic—injury, level of performance and BMI [61].

Lesser experienced climbers are experiencing more chronic injuries than elite climbers [58].

## Results—airborne sports

### Epidemiology—Paragliding

Most accidents occur in alpine areas, usually due to weather conditions [62]. Most accidents happen in May, July and August [63].

A study by Backer et al. showed that most airborne sport injuries were caused by paragliding (79.6%), followed by skydiving (10.5%) and Base jumping (6.1%) [64]. There is an injury rate of 10.8 injuries per 1000 participants per year for all different types of paragliding [65].

The mortality rate is about 0.46 per 1000 persons at risk [20].

The mean age at injury was 30.7–44.5 years [63–67]. As previously described, there is a higher preponderance for injury in males than females [63, 64, 67].

### Sites and types of injury—paragliding

Airborne sport injuries mainly involve the lower extremities, as seen in Table 3 [65, 67], especially the ankle joint [62]. Studies from Krüger et al. and Backer et al. showed similar injury frequencies of the lower extremities and the spine [64, 68].

**Table 3:** Injury region of injuries in airborne sports, whereas “n.r.” means not reported, “m:f” means male:female

Author	Investi-gation period	m:f (%) mean age	Number of injuries	Number of injured riders	Amateurs/Elites	Type of sports	Injury location							
							Upper extremity			Lower extremity			Trunk & Spine (%)	Head & Neck (%)
							Shoulder (%)	Hand/finger (%)	Upper/lower arm (%)	Hip/thigh (%)	Knee/calf (%)	Foot/ankle (%)		
Feletti et al. [66]	1995—2012	n.r. 44.5	256	n.r	Amateurs and elites	Parag Gliding	12.5	15.6	16.4	8.2	14.1	12.9	13.3	7
Feletti et al. [65]	2000—2014	88:12 n.r	1411	n.r	Amateurs and elites	Para Gliding	5.3	6.6	9.3	5.5	13.4	12.4	24.5	8.4
Cevik et al. [11]	n.r	75:25 n.r	17	20	Amateurs and elites	Para Gliding	17.6	35.3	17.6	11.8				
Fasching et al. [63]	1987—1991	92:8 33	n.r	70	Amateurs and elites	Para Gliding	12.8	32.5	39.3	15.4				
Mei-Dan et al. [9]	2006—2010	87:13 31	44	n.r	Amateurs	Base jumping	0	54.5	34.1	11.4				
Hasler et al. 2012 [59, 73]	2000–2009	91:9 n.r	11	11	Amateurs	Base jumping	54.5	9	9					
Monasterio et al. [10]	n.r	97:3 34	48	n.r	Amateurs	Base jumping	14.6	58.3	25	2.1				
Westman et al. [81]	1999–2003	70:30 n.r	311	257	Amateurs and elites	Skydiving	6.1	8.7	4.2	7.1	23.2	15.1	20.3	7.1

Feletti et al.'s study showed that the upper limbs were the most affected body area at 44.5%, followed by the lower limbs at 32% of injuries [66].

About 9.8–17.6% of the injured pilots had fractures of the spine [11, 65]. There is a high reported incidence of thoracolumbar fractures (Th11-L3) in the literature (74%). The most common fracture was an L1 fracture (30%) [64, 67]. In a study from Backer et al., the most prevalent Magerl classification type of fracture was Type A (91.5%) (compression Type), followed by Type B at 3.2% (Flexion-/Distraction Type), and type C at 5.3% (Translational Type) and pelvic ring fractures representing 9.4%. 4.4% had a spino-pelvic dissociation. Furthermore, multivariate analysis adjusted for age and gender showed a 21-fold higher Odds Ratio (OR) (OR 21.04, 95% Confidence Interval (CI) 7.83–56.57, *p* < 0.001) for spinopelvic dissociation fractures in paragliders than in the general trauma population [64]. There was a high prevalence of neurological signs ranging in most studies between 15.4% and 70.3% [62, 64] or even 100% in a study of Rekand et al. (n = 9) [67]. This is consistent with another study which found that pilots with spinal injuries resulting from crashes landed in 59% on their back or their buttocks [62].

The collapse of the airfoil during flight was the most common cause of accident (32.5%), followed by an uncontrolled crash-landing [62].

Other studies showed that especially during landing accidents happen (88.9%) [11, 69].

There was no statistically significant correlation between the severity of injuries and the flight experience of the pilots. Interestingly, 70.5% of the accident occurred in a topologically flat area [66]. Most crashes were caused by collision with a terrain feature [62, 66]. Many studies show that the take-off and landing is the most dangerous phase of flight [62, 63, 66]. Beginners have more accidents during take-off and landing, whilst experienced pilots are more accident-prone during flight [11, 62, 63].

### Epidemiology – Base jumping

Participants commonly jump from cliffs or buildings. 97% of participants who suffered fatal injuries were male. Almost all fatal injuries occurred between April and October. The injury rate of base jumping is lower than expected at a rate of 2 severe injuries per 1000 jumps (0.2% severe injury rate). The median age was 31 years [9]. Mostly these are men [70]. The fatality rate is about 0.4 deaths per 1000 jumps [70]. The most prevalent factor leading to fatality was low pull (deploying the parachute at an insufficient height for full deployment) and no pull [71].

The majority of injuries occurred from April to October [72].

Most injuries are related to object strike and bad landings [70].

### Sites and types of injury – Base jumping

The typical injury sites were the lower limbs, followed by the trunk and the spine. Head injuries occurred relatively rarely, as seen in Table 3 [9, 10, 73]. Total time base jumping correlates with injury frequency [9].

The lower limbs can be involved in landing accidents, as well as non-accidental fall events. The feet-first-position is the landing position that most commonly results in survival in falls from heights [74].

A study from Baecker et al. showed a high number of Spinopelivc dissociation in airborne sports. In their series, the prevalence was 36.4% and 21.1% occurred because of base jumping [64].

More than half of patients have multiple body injuries (55%) [72].

16% of accidents with fatal injuries were related to the use of wingsuits, primarily because of flight path miscalculation [75]. There also tended to be lower flight speed, leading to less aerodynamic control and less stability [10]. Soreide et al. showed that only 0.04% of all the jumps were fatal. The number of accidents increased with the number of jumps, but fatal injuries did not increase. Most non-fatal accidents resulted in injuries such as ankle sprains/fractures, minor head concussions, or bruising [76].

### Epidemiology—Skydiving

Skydiving has an injury rate of 17.4/10.000 skydives. Most injuries were minor. More significant injuries (requiring emergency room presentation) occurred at a rate of 6.0/10.000 skydives [77]. More men than women get injured (69%) [78].

The death rate is about 0.57 per 100,000 jumps [78].

Four out of five (83.3%) of the injuries occurred during the landing phase [78, 79], with a further 7.6% during freefall, 4.5% on parachute opening, 1.8% on aircraft exit, 1.2% during parachute flight, and1.7% before or after the jump. Almost half of the injuries (42.1%) occurred during the skydivers’ first ten jumps. [78]

Miscalculations during wing parachute flight and turbulence were major risk factors [80]

### Sites and types of injury—Skydiving

Student skydivers had the highest fatality risk, often caused by instability during freefalling leading to unstable parachute activation with subsequent line entanglement. One-third of all fatal injuries had an inflated parachute at the time of the accident [81].

As seen in Table 3, most time, the lower limbs were injured, especially the knee and calf [80]. This is followed by the injuries of the spine and trunk [80].

### Prevention – Airborne sports

Only one study was found that discussed injury prevention strategies. Schulze et al. assert that foam multi-chamber and airbag harnesses are the most effective precautions against spinal and pelvic fractures due to their shock-absorbing properties. Flying with an experienced trainer also effectively minimizes injury [62].

## Discussion

There is literature exploring medical aspects of injuries in alpine summer sports like mountain biking, climbing and airborne sports. Nevertheless, there is no single article providing a general overview, as individual studies focus on one particular sport.

By stark contrast to winter alpine sports, summer sports have been under-investigated, astonishing given their increasing popularity, particularly hiking [2]. We wanted to dedicate a chapter giving an overview of the injuries suffered whilst hiking in the Alps but studies were so scarce that we refrained from the idea. However, the Swiss Alpine Club (SAC) showed that between 2014 and 2016 most injuries requiring rescue and hospitalization occurred whilst hiking (n = 1007 in 2014, n = 1193 in 2015, n = 1195 of 2828 patients in 2016). Hiking also had the highest fatality rate between 2015 to 2019 of all recorded types of sports the alps [1, 2]. Because of that, further investigation into injury patterns and possible prevention mechanism is needed.

Similarly, water sports such as canyoning and rafting related injuries are rarely described in the literature [82]. Despite increased popularity over the last 20 years with high-energy trauma mechanisms and reported fatal injuries, we found only two studies from Murdoch et al. and Ströhle et al. which described the most common regions of injuries and the injury mechanism in rafting and canyoning [82]. But overall, there has been no high-quality study evaluating precipitating factors as the time of year and weather conditions in predicting risk of injury.

Male summer alpinists were more commonly injured and susceptible to accidents than women [9, 18, 21, 22, 41, 42, 44, 45, 63–65, 67, 68, 77]. This finding may be confounded because males who are arguably more prone to risk-taking behaviour most often undertake these sports.

Concerning prevention, few studies analyzed the effectiveness of helmet use, airbags, back guards, multichambered foams or finger-tapping, especially in sports like climbing, paragliding, skydiving and base jumping. In contrast to alpine winter sports, where 12 meta-analyses have shown that wearing a helmet significantly reduces the risk of head injuries [83], in mountain biking there is still some controversy regarding the benefit of helmet use [17, 21]. The interplay between the safety conferred by the safety device versus a false sense of security potentially influencing risk-taking behaviour would be particularly interesting.

Currently, there are only four larger surveys analyzing summer alpine sports. These are Feletti et al. [66] and Feletti/Aliverti et al. [65] in airborne sports and Schöffl et al. [43] regarding climbing, as well as Kotlyar et al. [22] concerning mountain biking. As a result, information surrounding injury mechanism, sites and types, risk factors, epidemiology of injured patients and preventive methods is relevantly limited. For a better understanding of injury types and fracture patterns regarding outcome, it would be beneficial to conduct prospective studies.

This review is limited by the heterogeneity of studies regarding alpine sports in summer. There are rarely high-quality studies with control groups reporting any outcome. Another limitation is that the included studies were not all sufficiently indexed. This again is related to the sparsity of available data. The included studies also had individual limitations, including publication bias and wildly varying data collection and presentation (for example, non-uniform localization subdivision).

## Conclusion

Mountain biking, climbing, or aerial sports involve a wide range of injury sites and mechanisms. Mountain bike-related injuries usually involve the upper extremity, as the most common accident mechanism is a fall over the handlebars. Here, fractures of the clavicle occur as the most common injury, followed by fractures of the hand and wrist. A helmet may reduce injuries of the head and face as well as the need for neurosurgical consultation.

Climbing injuries often involve the extremely exposed hand, especially the flexor pulley system. In this context, injuries to the lower extremities most often affect the foot and ankle, with the cause usually falling from high altitudes.

In paragliding, skydiving and base jumping, the transitional areas of the spine such as the thoracolumbar and spinopelvic regions are particularly affected. Injuries to the lower limbs have been observed as frequently as injuries to the spine. The cause of the injuries are usually problems with landing or falls.

In terms of relative risk, mountain biking has the lowest risk, followed by climbing and aerial sports. Male alpinists seem to be more prone to injuries than female alpinists.

All in all, it can be summarized that specific injuries occur for each sport, depending on the mechanism of the accident. With this knowledge, especially in diagnostics and clinical management can be simplified and thus the overlooking of injuries can be avoided.

Regarding preventive possibilities, there is an apparent reduction of head and facial injuries when wearing a helmet in mountain biking and climbing. Regarding airborne sports, wearing foam multi-chamber and airbag harnesses have been identified as preventive. However, further work is needed to clarify the injury mechanisms and effective preventive measures.

## Data Availability

The datasets generated and analysed during the current study are not publicly available as a supplementary file because we share the algorithm for our data extraction strategy and therefore grant reproducibility. Data sets are available from the corresponding author on reasonable request.

## Abbreviations

SAC: Swiss Alpine Club
ISS: Injury Severity Score
n.r.: Not reported
